# Biological Traits and Genetic Relationships Amongst Cultivars of Three Species of *Tagetes* (Asteraceae)

**DOI:** 10.3390/plants11060760

**Published:** 2022-03-12

**Authors:** Raluca Cicevan, Adriana F. Sestras, Mariola Plazas, Monica Boscaiu, Santiago Vilanova, Pietro Gramazio, Oscar Vicente, Jaime Prohens, Radu E. Sestras

**Affiliations:** 1 Faculty of Horticulture, University of Agricultural Sciences and Veterinary Medicine of Cluj-Napoca, 400372 Cluj-Napoca, Romania; raluca.cicevan@gmail.com (R.C.); rsestras@usamvcluj.ro (R.E.S.); 2 Institute for the Conservation and Improvement of Valencian Agrodiversity (COMAV), Universitat Politècnica de València, Camino de Vera s/n, 46022 Valencia, Spain; maplaav@btc.upv.es (M.P.); sanvina@upvnet.upv.es (S.V.); ovicente@upvnet.upv.es (O.V.); 3 Mediterranean Agroforestry Institute (IAM), Universitat Politècnica de València, Camino de Vera s/n, 46022 Valencia, Spain; mobosnea@eaf.upv.es; 4 Instituto de Biología Molecular y Celular de Plantas, Consejo Superior de Investigaciones Científicas-Universitat Politècnica de València, Camino de Vera s/n, 46022 Valencia, Spain; piegra@upv.es

**Keywords:** seed characteristics, germination percentage, phenological stages, morphological traits, phylogenetic relationships

## Abstract

Marigolds (*Tagetes* spp.) are multifunctional flowering plants belonging to the Asteraceae family, well-known and widespread for their ornamental value and many other uses. In this study, morphological differences and genetic relationships among 21 cultivars of three species of marigold (*Tagetes patula*, *T. erecta* and *T. tenuifolia*) were analysed. Results have revealed obvious differences among genotypes, starting from the morphological characteristics of the seeds and their capacity to germinate to adult plant morphological characteristics, both between cultivars and species. The genotypic differences were manifested in considerable variation in the development of phenological stages and the main morphological traits of plants and flowers. PCA and hierarchical clustering analyses of morphological traits revealed a homogeneous grouping of cultivars within each species, except for Orion, belonging to *T. patula*, which was closer to *T. erecta* cultivars. A subset of 13 cultivars from the three species was subjected to SSR analysis, revealing considerable genetic diversity and good separation between *T. patula* on the one side and *T. erecta* and *T. tenuifolia* on the other. The observed heterozygosity was much lower than the expected heterozygosity, revealing a high degree of fixation. The results reveal that the three species evaluated have considerable morphological and genetic diversity, which has important implications for assessing genetic diversity, conserving germplasm and selecting parents for new breeding works in marigolds.

## 1. Introduction

The genus *Tagetes*, which includes plants known as ‘marigolds’, belongs to the Asteraceae family and contains more than 50 cultivated and wild species. Marigolds are native to America, but several species are naturalised in Africa, Asia, and Europe [1,2]. Plants of the genus *Tagetes* are amongst the most widespread garden flowers worldwide. Marigolds are popular amongst gardeners due to their easy cultivation, wide adaptability, low demands for ecological and technological factors, and flower production throughout the year [3].

As ornamental plants, marigolds have several advantages through which they have become particularly appreciated and widely cultivated in the world. These include: their suitability for a large type of cultivation, i.e., for bedding, edges, and pots, but also as cut flowers, bouquets, different floral arrangements, and other applications recognised in social or even religious life; long flowering period extending throughout the summer and fall; easy reproduction by seeds; and the great diversity of varieties within several species of *Tagetes* [3,4,5]. Furthermore, due to their multiple utilities, the short production period of decorative and marketable flowers, a broad spectrum of attractive colour palettes, shapes, and sizes of flowers, and also plant habits, marigolds have become one of the preferred species of many amateur and commercial flower growers [6].

As a crop species, with a short period necessary for cultivation, marigolds can be grown in a multi-crop system, rotated with other crops, or as a mixed crop. In the latest case, cultivated on the borders with other plants can assure beneficial effects in organic agriculture management [7], mainly in the production of important horticultural species, e.g., tomatoes [4]. Due to their bactericidal, nematicidal, fungicide and insecticide action, *Tagetes* species can be used in organic agriculture, especially in the culture of vegetables. Thiophenes, alkaloids, polyacetylenes, fatty acids, flavonoids and terpenes compounds present in *Tagetes* confer antimicrobial and nematicidal effects, which may be of interest in organic farming. The use of natural or biological origin pesticides has the advantages of low toxicity, efficient control, and reduced cost. In addition, these products do not promote resistance to pests and diseases in agricultural crops [7]. Highlighting the role and importance of *Tagetes* plants in Mexican culture, Tapia-Vázquez et al. [8] note their widespread use as an antioxidant, medicine, food pigment, flavouring, perfume, resin, ornamental and insecticide. Biocidal extracts from roots, stems, leaves, inflorescences, or from the whole plant allow wide use in agriculture as a nematicide, larvicide, attractant or insecticide and as a fertilizer [4,9]. *Tagetes* can be used in crop rotation and, in pest control, can be applied as organic manure to plants or as aqueous extracts and powders [8]. The variation in *Tagetes* cultivars belonging to different species allows the identification of genotypes with resistance or tolerance to saline and drought stresses and other adverse conditions [4,10,11].

Marigolds are widely used for industrial and medicinal purposes and also as natural dyes and insecticides or herbicides [4,12,13,14,15]. Due to their importance as medicinal plants in health care and herbal preparations, marigolds are cultivated and produced at an industrial scale, particularly in developing countries from the Far East. In addition, marigolds have received increasing attention in scientific research because of their multiple uses and their phytochemistry and bioactivity importance [6,16]. However, marigolds are extremely appreciated as annual ornamental plants, *Tagetes patula* L., *T. erecta* L. and *T. tenuifolia* Cav. being amongst the best known and most widespread. In addition to their value as ornamental plants, these three species have diverse and extremely numerous uses and properties, making them very interesting species to be used in marigold breeding works [17].

The species *T. patula* is native to the highlands of Mexico and Argentina but is now widespread throughout the world, known as the French marigold. The date of its introduction into Europe is uncertain, but it was first reported in France around 1880 [18]. The species is also well acclimatised in Romania, where it is widely cultivated in gardens and green spaces. The plants of this species are small, about 40 cm, and have strong branches and elongated leaves (dark green), with pinnate compositions. Their floral heads are small, with thick peduncles, and the flowers are usually hermaphroditic, yellow, orange or a combination of colours. Flowering takes place gradually, starting in July and extending until September.

*Tagetes erecta* originates from Mexico and was first introduced to Europe in Spain in the 16th century [19,20]. It is known as the African marigold, Aztec marigold, American marigold, or big marigold. The species is well known in Romania. In Mexico, the species can reach up to 1.5 m or more, but the average of the most commonly used varieties is 60–80 cm [21]. Excessive growth often complicates its growth in pots. For this reason, new commercial varieties have a height of only about 25–38 cm [21]. The stems are glabrous or pubescent with green branches and numerous long ribs. The leaves are pinnately compounded. It blooms from July to October. The flower heads are large, with a diameter of 4–7 cm, comprising many individual flowers, usually orange in colour [21]. *Tagetes erecta* has multiple uses as an ornamental plant in decorating green spaces and various floral arrangements and garlands; it is planted in crops as an insect repellent and has use in pharmacy and medicine, industry, and so forth [22]. African marigold meal and extracts are used in poultry feed to colour the skin, meat (fat) and egg yolks and as colourants in a wide variety of food products [23]. Yellow-orange flowers are sources of great interest in the extraction of natural oils and pigments. Lutein is extracted from flowers, the main pigment used to produce the colour orange, *T. erecta* being the main source of this pigment for commercial uses [20]. The species can be used as natural air filters or sinks to alleviate air pollution [24]; it also has a strong invasive potential [20] and, from cultivated gardens, can easily migrate into spontaneous flora and spread freely, Romania being recorded as being invaded by the alien flora [25].

The origin of *T. tenuifolia* is found in America, and it occurs from Mexico throughout Central America to central Colombia [26]; compared to the two previous species, it is less common. It is known as signet marigold, golden marigold or lemon marigold. It has smaller dark green compound leaves and smaller, yellow, orange, golden or multicoloured floral heads. It gradually blooms from June to October [27,28].

Marigold seeds are usually sown directly in the field [29]. The optimal sowing period varies depending on the geographical area. In Romania, this period corresponds to mid-April. Seed germination is influenced by various environmental factors, especially soil temperature and humidity, as well as watering frequency and water quality used. Seed quality is paramount in marigolds to achieve rapid and uniform seedling emergence, avoid gaps in the field and ensure uniform field culture [30].

There are different studies of morphological and genetic diversity within and among species of *Tagetes*. The knowledge of morphological and decorative characters or useful biochemical components and their genetic variability could support new breeding programmes for different species of interest [31,32,33,34,35] as well as for marigold [36]. Genetic variation and relationships among genotypes are essential in classifying and utilising germplasm resources in marigold breeding [37]. Using RAPD markers in the fingerprinting of marigold, 22 cultivars of *T. patula* were considered a suitable reservoir of useful genes that could be utilized for interspecific and intervarietal crosses to obtain variation, which can be exploited to create new valuable ornamental cultivars [38]. In another study, both RAPD and STS markers were successful in estimating the genetic diversity of *Tagetes* species and genotypes (*T. patula*, *T. erecta*, *T. minuta*) and in selecting parents for breeding and mapping purposes [39]. SSR markers also proved effective for genetic diversity analysis, species classification and individual identification in commercial cultivars and landraces of *T. patula* and *T. erecta* [40]. The trends in modern plant breeding programmes may have contributed to the genetic vulnerability of all cultivated species of *Tagetes*. Moreover, the genetic background of new cultivars in marigolds must be diversified to reduce the risk or possible crises in the future and minimise vulnerability. New hybrids possessing an intense heterosis effect, with superior ornamental traits and market values, can be produced using interspecific hybridisation, e.g., between African marigolds and French marigolds [41,42]. In this way, the success of new breeding programmes in *Tagetes* depends on the genetic variation of the germplasm as well as on its efficient use [43].

Based on the above considerations, this study aims to characterise several cultivars of *T. erecta*, *T. patula* and *T. tenuifolia*. Information on seed characteristics was analysed in relation to germination capacity and seedling appearance. Another question addressed in this study was to assess how these characteristics affect the growth and vegetative development of plants and their reproductive efficiency (number of flowers and set of seeds). The analysis of the plants’ phenophases of growth and fruiting and the main traits of the plants was performed comparatively both inside the species (within the tested cultivars) and between the species. Furthermore, the genetic variation and phylogenetic relations of the cultivars were investigated with SSR markers.

## 2. Results

### 2.1. Seed Characteristics

Both for the main characteristics of the seeds and the germination of the seeds, there were obvious differences between the analysed varieties (Table 1). In *T. patula*, the seed length ranged between 8.8 mm (Bolero) and 14.0 mm (Orion). The trait variation amongst the nine cultivars of the species was very different, with values of the coefficient of variation between 1.1% (Queen Sophia) and 22.3% (Bolero). Significant differences between cultivars were also recorded for the width and thickness of the seeds. The best germination capacity of the seeds was recorded in the Orange Flame, Robuszta and Orion cultivars (all with 100% germinated seeds). Instead, the seeds of the cultivar Mr. Majestik had low germination (43.5%). In the five cultivars of *T. tenuifolia*, the amplitude of variation for seed traits was much lower (e.g., between 6.6–8.8 mm for seeds’ length), as was the germination of seeds (this varied between 87.0% in Sarga and 100.0% in Luna Gold and Luna Lemon), although significant differences between cultivars were detected. In *T. erecta*, the seed length ranged between 10.9 and 15.1 mm, these extreme values being obtained on Aranysarga and Antigua Orange cultivars, respectively. For all seed traits, there were significant differences between cultivars. Seed germination was 100% in a single cultivar (Magas Citromsarga) but low in Alacsony Citromsarga (65.2%) and extremely low in Arctic (8.7%).

The variation of traits of seeds within the varieties was generally small (CV% below 10%) in a few cases, registering values of CV% that indicated an intermediate (CV% between 10.1–20%) or high (CV% over 20.1%) variation. The last category included Bolero for seed length (CV% = 22.3) and Orange Flame, with CV% just over 20%, for seed width and thickness.

**Figure 1:**
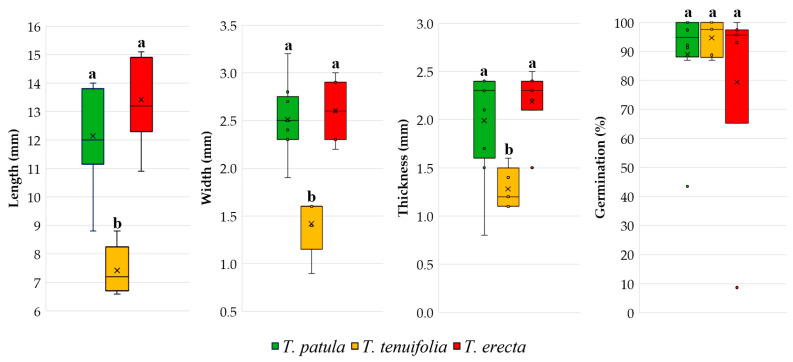
Main traits of the seeds based on the cultivars analysed in the three species of *Tagetes*. Each box plot represents minimum, lower quartile, median, upper quartile, and maximum values; in addition, the mean of the data is symbolised with a ‘✕’ in the boxplot. Different letters among boxplots indicate statistically significant differences for the respective trait, at a significance level of *p* < 0.05 (Duncan test).

Boxplots and Duncan multiple range tests (Figure 1) highlight the differences between the three species of *Tagetes* for the three main traits of the seeds. Between *T. patula* and *T. erecta*, there were no significant differences between the means of seed length, width and thickness. In contrast, the mean values of these traits in *T. tenuifolia* were significantly lower than those of the first two species mentioned. Only for seed germination, *T. tenuifolia* does not differ significantly from the other two species, *T. patula* and *T. erecta*. The graphs also suggestively indicate the dispersion of data within and between species. For the characteristics of the seeds, the whiskers of the boxplots suggest a large dispersion of the cultivar values in *T. patula*. Outliers or abnormal values, marked by small dots, appear only in *T. erecta* for seed thickness. Instead, such aberrant values are highlighted by boxplots for seed germination in both *T. patula* and *T. erecta*.

### 2.2. Plant Growth Traits

Significant differences between varieties within species were detected for plant height, stem diameter number of branches per stem, and angle of branches (Table 2).

The amplitude of the variation for plant height ranged between 25.4 and 47.8 cm for *T. patula*, 21.0 and 21.8 cm for *T. tenuifolia*, and 24.4 and 69.8 cm for *T. erecta*. The extreme mean values of the stem diameter were comprised between 8.5 and 12.9 mm (*T. patula*), 8.7 and 9.2 mm (*T. tenuifolia*), and 8.9 and 15.8 mm (*T. erecta*). The branching of the plants was very different, the average number of branches on the stem being between the following values: 7.6 and 13.7 (*T. patula*), 16.0 and 16.9 (*T. tenuifolia*), and 8.9 and 14.2 (*T. erecta*). Finally, the fluctuation of the average values for the insertion angle of the main branches oscillated between the following limits: 40.0 and 60.6 degrees (*T. patula*), 51.5 and 52.8 degrees (*T. tenuifolia*), and 37.3 and 71.1 degrees (*T. erecta*). 

The coefficients of variation of these traits generally had values appreciated as small (CV% below 10.1%) or even very small, i.e., for the angle of branch insertion, for all three species. 

**Figure 2:**
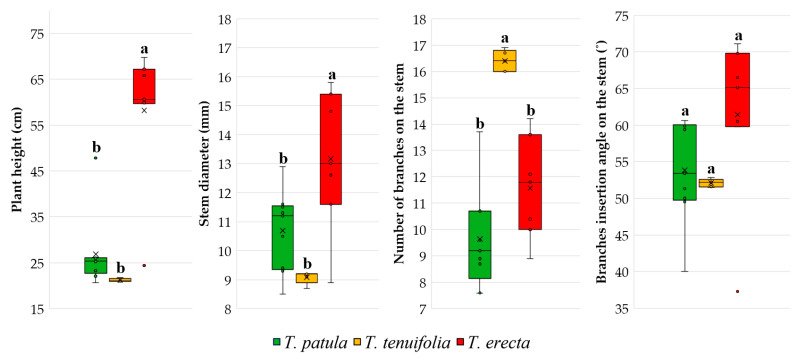
Main traits of the plants, based on the cultivars analysed in the three species of *Tagetes*. Each box plot represents minimum, lower quartile, median, upper quartile, and maximum values; in addition, the mean of the data is symbolised with a ‘✕’ into the boxplot. Different letters among boxplots indicate statistically significant differences for the respective trait at a significance level of *p* < 0.05 (Duncan test).

The boxplots with plant growth (Figure 2) evidenced a higher plant height of *T. erecta*, with a mean of cultivars close to 60 cm. Duncan’s multiple range tests indicated that the mean of the analysed cultivars of this species is significantly higher than the means of cultivars of *T. patula* and *T. tenuifolia*. In addition, it can be seen that the mean plant height of *T. erecta* is reduced by a value that the boxplot highlights as an outlier. From Table 1, it can be found that the respective cultivar is Antigua Orange, with an average plant height of only 24.4 cm, much lower compared to the other cultivars of *T. erecta* species. An opposite situation occurred in *T. patula*, in which an outlier (Queen Sophia cv., with 47.8 cm) raised the mean of the species.

For all the plant growth and branching characteristics, the boxplots indicate a fairly high homogeneity of *T. tenuifolia* cultivars. Of the three species, *T. erecta* stands out for its significantly higher differences in stem diameter and *T. tenuifolia* for the number of branches per stem. For the angle of branches insertion, there were no real differences within the three species.

### 2.3. Main Traits Related to Flowers and Fruits

For the traits analysed and presented in Table 3 (number of flowers per plant, corolla diameter, disc diameter, number of fruits), only in the case of disc diameter in *T. tenuifolia* species there were no differences between cultivars within the same species. A higher number of flowers per plant was recorded in *T. tenuifolia* (between 91.9 and 96.4), but a greater variation in trait per species was noted in *T. erecta* (5.3 and 32.7). The corolla diameter was small and uniform in the varieties belonging to the species *T. tenuifolia*, of medium size in *T. patula* (with an amplitude of varieties between 3.0 and 5.4 cm) and large in *T. erecta* (with limits between 6.4 and 10.1 cm). Disc diameter of the flowers was small and uniform in the cultivars of *T. tenuifolia* and *T. patula*, but large compared to *T. erecta* cultivars. The numbers of fruits (achenes) in the heads oscillated, depending on the species: *T. patula* (4.8 and 13.1); *T. tenuifolia* (32.1 and 36.0); *T. erecta* (2.6 and 10.9). For this last trait, the most significant variability was registered especially in the cultivars Robuszta, Cresto Yellow and Bolero (all of the *T. patula* species, with CV% over 20); Hawaii, Arctic, Aranysarga, and Antigua Orange (all of the *T. erecta* species; the first two with CV% over 30, and the next two over 20).

Amongst the main phenological phases (Table 4), seedling emergence showed a higher amplitude of the number of days required in *T. patula* (10.0–27.0), followed by *T. erecta* (14.0–24.0). A smaller variation was recorded for *T. tenuifolia* cultivars (13.0–15.0). Stem formation required a more variable number of days to *T. patula* (19–30 days) and *T. erecta* (29–39 days) cultivars, whereas in *T. tenuifolia* cultivars, this phase unfolded more homogeneous (22–25 days). The cultivars of *T. tenuifolia* also had the following vegetative phenophases (branching, budding) and were more uniform in their progress compared to *T. patula* and *T. erecta*. The cultivars of *T. tenuifolia* showed a great uniformity for developing the main generative phenophases: full flowering, flowering duration, fruit formation, and fruit ripening, respectively. Probably, this particularity was due to the group of cultivars from the ‘Luna’ range (four cultivars), with close origin.

The long duration of the number of days required from sowing to reach full flowering in some cultivars of *T. patula* was noted, as well as the variation of this trait between cultivars (41–100 days). Even in *T. erecta* (70–83 days), the number of days to reach full flowering was much higher than in *T. tenuifolia* (45–47 days).

Flowering duration, reported as an average of the number of days a flower maintains its decorative features at an appropriate level, was relatively close for the three species; the highest amplitude was obtained in *T. patula* cultivars (15–26 days). Regarding fruit formation and fruit ripening, the longest period required, but also the highest amplitude of the two characters, was recorded in *T. patula* (between 54–118 days for fruit formation and between 67–145 days for fruit ripening).

**Figure 3:**
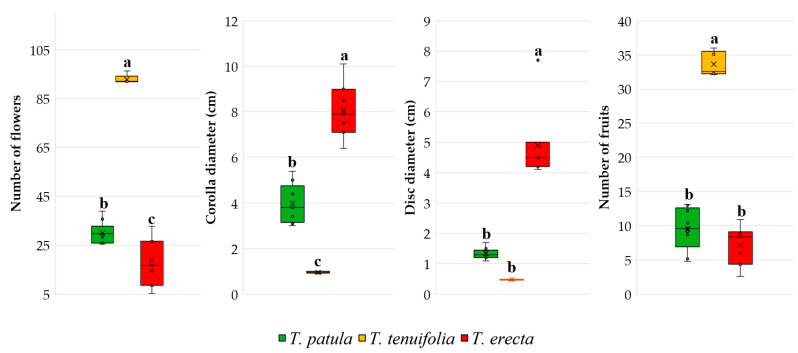
Main traits of the flowers and the number of fruits, based on the cultivars analysed in the three *Tagetes* species. Each box plot represents minimum, lower quartile, median, upper quartile, and maximum values; in addition, the mean of the data is symbolised with a ‘✕’ into the boxplot. Different letters among boxplots indicate statistically significant differences for the respective trait, at a significance level of *p* < 0.05 (Duncan’s test).

The species *T. tenuifolia* differs from the other two species for the main traits of the flowers and the number of fruits (Figure 3). Only for the diameter of the floral disc, *T. tenuifolia* does not differ significantly from *T. patula*. The boxplots and Duncan’s multiple range tests also highlight the high number of flowers and fruits in *T. tenuifolia* compared to the other two species and the extremely narrow variability of flower traits recorded in *T. tenuifolia*.

**Figure 4:**
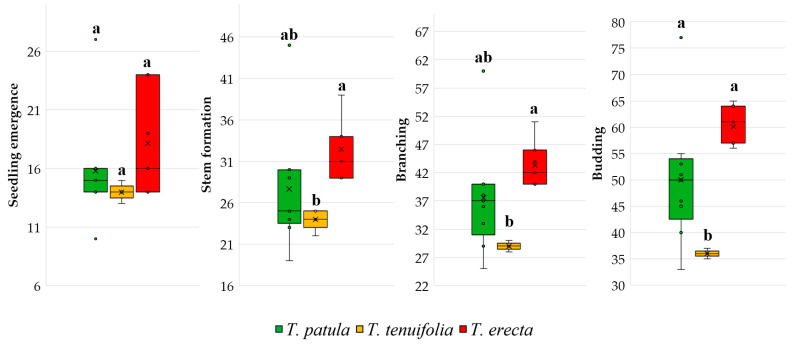
Main phenological stages based on the cultivars analysed in the three species of *Tagetes*. Each box plot represents minimum, lower quartile, median, upper quartile, and maximum values; in addition, the mean of the data is symbolised with a ‘✕’ into the boxplot. Different letters among boxplots indicate statistically significant differences for the respective trait, at a significance level of *p* < 0.05 (Duncan’s test).

In Figure 4, the range and distribution of the area for the vegetative phenophases of the three species of *Tagetes* are presented. There is a smaller variability for *T. tenuifolia* for all traits, whereas a more significant variability is highlighted for seedling emergence of *T. erecta*. In addition, the boxplots emphasise the apparition of some larger outliers of *T. patula*, visible for all four traits. Duncan’s multiple range tests showed that there were no significant differences between the three species for seedling emergence; instead, such differences were recorded for stem formation, branching and budding.

**Figure 5:**
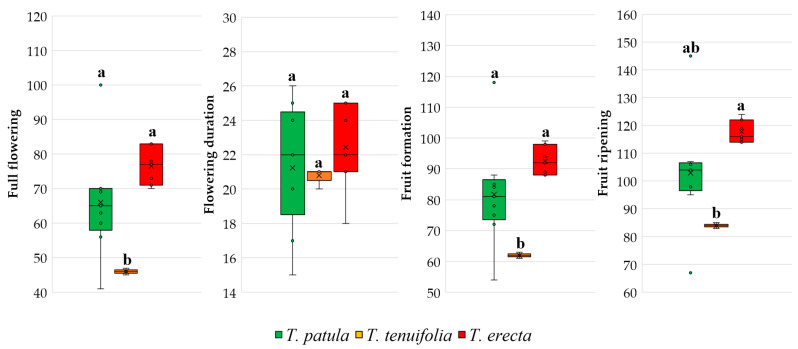
Main reproductive traits and phenophases based on the cultivars analysed in the three species of *Tagetes*. Each box plot represents minimum, lower quartile, median, upper quartile, and maximum values; in addition, the mean of the data is symbolised with a ‘✕’ into the boxplot. Different letters among boxplots indicate statistically significant differences for the respective trait at a significance level of *p* < 0.05 (Duncan’s test).

Regarding the main generative phenophases, the lowest variability was also recorded in the *T. tenuifolia* species (Figure 5). Even if there were no significant differences between the three species for the duration of flowering, unlike *T. tenuifolia*, in which the cultivars showed a remarkable uniformity of flowering period, in *T. patula* and *T. erecta* cultivars, the flowering period was longer. Boxplots suggestively differentiate the dispersal of flowering days in the last two species, respectively, by the trait variation, depending on the cultivars’ genotypes.

### 2.4. Phenotypic Correlations between the Analyzed Traits

By calculating the phenotypic correlation coefficients between the analysed traits, numerous positive or negative relations, statistically significant as *p* < 0.05, 0.01 and 0.001 (Table 5), were highlighted between different traits of seeds, plants, flowers, or the vegetative and reproductive phenophases, fruit formation or seed ripening, respectively. 

Thus, significant positive correlations were detected amongst seed traits (length, width, and thickness). In addition, these were positively correlated with corolla diameter and disc diameter, stem formation, branching, budding, and also with full flowering, fruit formation and fruit ripening. On the contrary, the three seed traits were negatively correlated with the number of branches per stem, the number of flowers, and the number of fruits.

Amongst some elements of plant vigour, such as plant height and stem diameter, there were positive correlations with decorative elements of flowers (i.e., corolla diameter, disc diameter, full flowering), or even with fruit formation and fruit ripening. However, there were also some inversely proportional relations (negative correlations) with the number of flowers and fruits. The emergence of seedlings was closely correlated with stem formation, branching, budding, full flowering, flowering duration, fruit formation, and fruit ripening. Generally, significant, positive correlations were identified amongst all these characters. 

It is interesting that between a trait of great ornamental interest, such as the number of flowers, significant negative correlations were registered, some of them unexpected, with numerous analysed traits, for example, the morphological particularities of the seeds (length, width and thickness), plants (height and stem diameter), flowers (corolla diameter and disc diameter), or vegetative and reproductive phenophases (stem formation, branching, budding, full flowering, fruit formation and fruit ripening). Contrary to these negative correlations, the number of flowers was positively correlated with the number of branches on the plant and the number of fruits. The correlations registered between the number of flowers and different traits analysed were quite close also for the number of fruits (as the direction, positive or negative, and significance of correlations). Unlike the number of flowers, the corolla diameter and the disc diameter were positively correlated with most of the traits, except for the number of flowers and fruits.

### 2.5. Multivariate Analysis of Traits (Principal Component Analysis) and Genetic Variation

**Figure 6:**
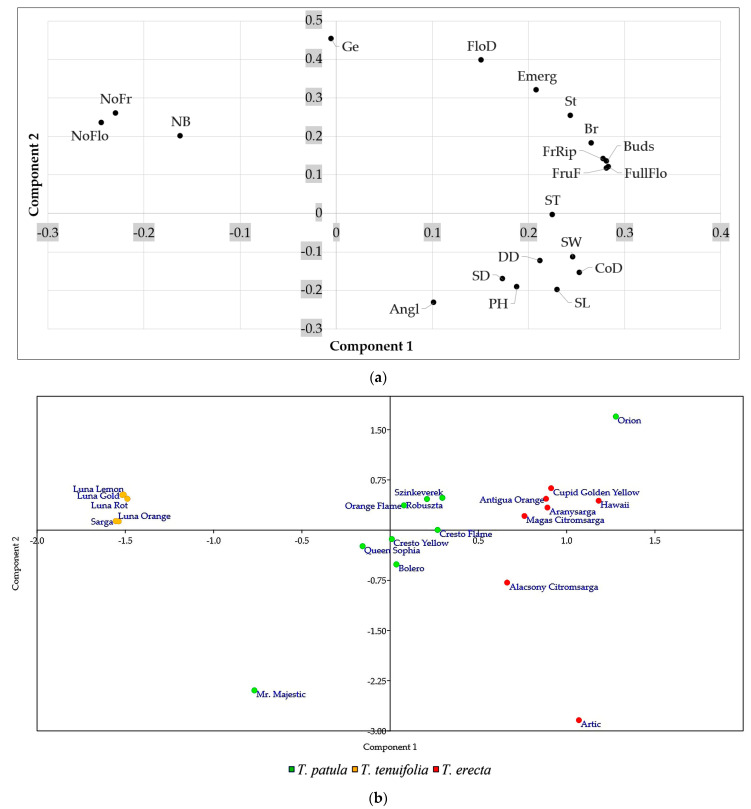
Principal component analysis (PCA) plot: (**a**) the multivariate variation of the traits of the 21 cultivars belonging to three *Tagetes* species (*T. patula*, *T. tenuifolia* and *T. erecta*); (**b**) the multivariate variation of the 20 analysed traits: SL (seed length); SW (seed width); ST (seed thickness); Ge (germination); PH (plant height); SD (stem diameter); NB (no. branches/stem); Angl (angle of branch insertion); NoFlo (no. of flowers); CoD (corolla diameter); DD (disc diameter); NoFr (no. of fruits); Emerg (emergence); St (stem formation); Br (branching); Buds (budding); FullFlo (full flowering); FloD (flowering duration); FruF (fruit formation); FrRip (fruit ripening).

The principal component analysis (PCA) plot provides an interesting visual projection and essential information of the relationships between the three species of *Tagetes* based on the multivariate variation of the 20 analysed traits (Figure 6a). The PCA illustrates a compact grouping of variables, which are grouped together and appear as positively correlated (i.e., fruit ripening, fruit formation, budding, full flowering, branching—in quadrant I; seed width, seed length, seed thickness, stem diameter, plant height, corolla diameter, disc diameter—quadrant II).

Also closely grouped and positively correlated are the number of flowers and fruits and the number of branches per stem, all situated in quadrant IV. Their opposite positioning (and negative correlations) can be seen compared to the traits in quadrant II, including the branching angle of the main branches on the plant. PCA confirms the relations identified with Pearson correlations and may suggest that the fastigiate growth of bushes and branches with a basitone tendency is in a relatively inverse proportion to the abundant flowering and fruiting capacity. 

The PCA of the 21 cultivars (Figure 6b) highlights that the cultivars of *T. tenuifolia* are very close to each other and form a particularly homogeneous group, contrasting with some cultivars of *T. patula* (e.g., Bolero, Cresto Flame, Cresto Yellow) or *T. erecta* (Alacsony Citromsarga). Some of the *T. erecta* cultivars are located quite compactly in quadrant I, but two are included in quadrant II, one even at a great distance (Arctic). In the *T. patula* species, most cultivars are grouped close to the centre, respectively, to the origin of the plot. This could indicate that they have average properties, but this assertion is contradicted by the opposite positioning towards the extremities of the two opposite quadrants of Mr. Majestic and Orion cultivars.

The first and second components of the PCA accounted for 62.19% and 20.28%, respectively, of the total variation observed.

**Figure 7:**
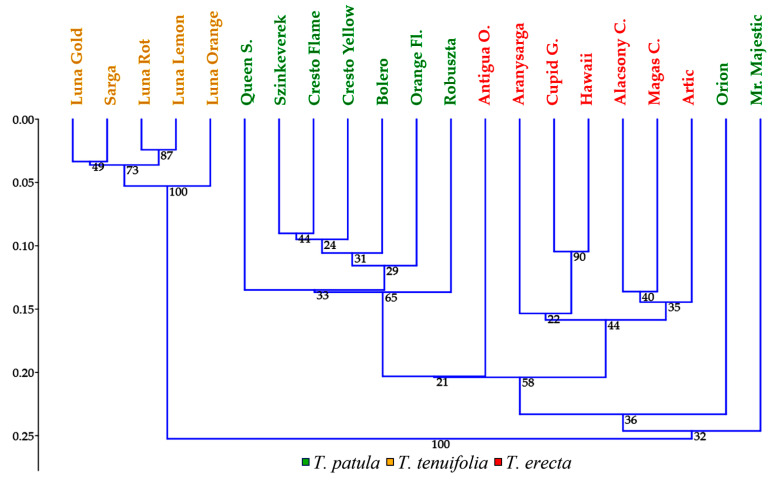
Hierarchical clustering analyses of the 21 cultivars belonging to three *Tagetes* species based on 20 morphological traits; single linkage method, Gover similarity index.

Hierarchical clustering analyses illustrated a homogeneous grouping of cultivars within each species of *Tagetes* (Figure 7). Only one exception was recorded for the Orion and Mr. Majestic cultivars, belonging to *T. patula*, which were situated on two different clusters, clearly spaced from the compact group of the other species cultivars (these being placed on a common subcluster).

Based on the phenotypic traits and phenological attributes conferring general peculiarities of greater ornamental and landscape importance, 13 cultivars from the three species were subjected to molecular analysis. The SSR technique was considered optimal for phylogenetic analysis and for testing the genetic diversity among cultivars.

In order to assess the genetic diversity present amongst the different *Tagetes* genotypes, a set of six SSRs developed by Whankaew et al. [40] were selected, taking into account the number of alleles detected and the PIC (Table 6). A total of 13 alleles were detected using six SSR combinations with a mean of 3.5 alleles per locus. Although the expected heterozygosity has a mean of 0.46, the observed heterozygosity was very low in general, ranging from 0.00 to 0.23. This indicates a high degree of homozygosity, probably due to the various floral mechanisms adapted in Asteraceae to reduce the likelihood of self-pollination within co-sexual flowers [44]. The PIC (polymorphic information content) value, which assesses the informativeness of the markers in a population, ranged from 0.18 in the marker TE11, which was much lower than the others, to 0.66 in the marker TE59, with a general mean of 0.40.

Similar results were observed by Whankaew et al. [40], analysing the genetic diversity of 40 individuals of two different *Tagetes* species (*T. erecta* and *T. patula*). This work detected a mean of three alleles per locus, with an expected heterozygosity average value of 0.48, and the markers had an average PIC of 0.48. The observed heterozygosity was higher (0.32) than that observed here, most likely due to the inclusion of commercial hybrids in their study.

**Figure 8:**
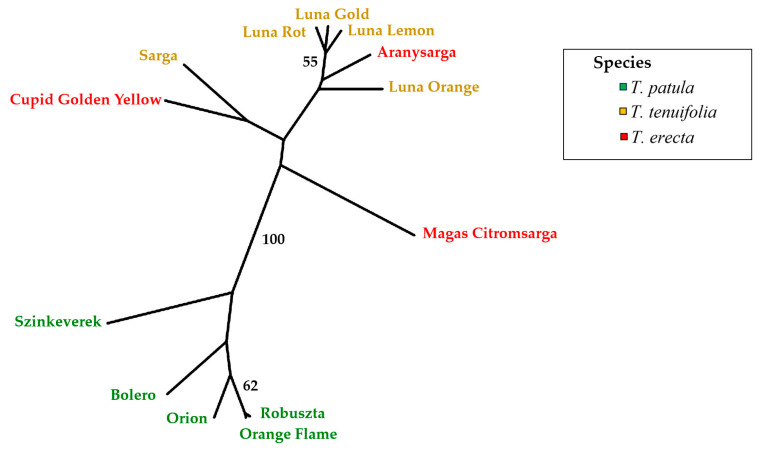
Unrooted UPGMA phenogram of 13 *Tagetes* cultivars based on six polymorphic SSR markers. The species of each cultivar is identified with a different colour.

The UPGMA dendrogram using Nei’s genetic distance [45] distributed the three species into two main clusters (Figure 8) based on 1000 bootstrap randomisations. The first cluster separates all *T. patula* cultivars from the rest with a bootstrap value of 100%. The second cluster is composed of the rest of the individuals belonging to the other two species. No clear separation by species was observed in this cluster, although all ‘Luna’ cultivars (*T. tenuifolia*) clustered together with the *T. erecta* cultivar Aranysarga. The pairwise genetic distance values (Table S1) confirm the results obtained with the UPGMA dendrogram. In this way, genetic distances within *T. patula* were generally lower than the genetic distances with cultivars from the two other species, and *T. tenuifolia* ‘Luna’ cultivars also displayed low genetic distances among them. 

**Figure 9:**
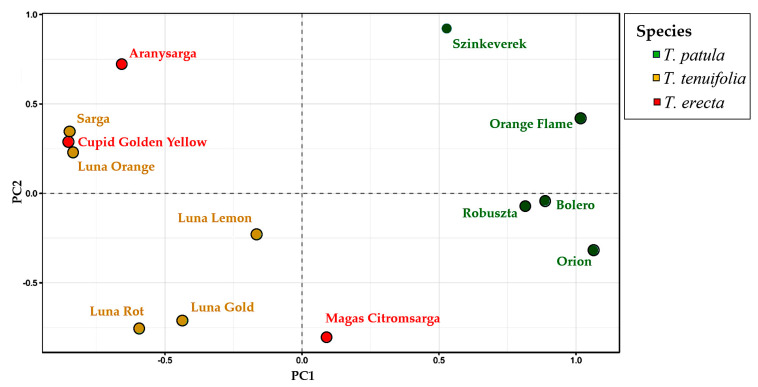
Relationships between the 13 *Tagetes* cultivars based on six SSRs according to the first and second principal coordinates obtained from a principal coordinates analysis (PCoA). The first and second principal coordinates account for 33.75% and 17.61% of the molecular variation. The species of each cultivar is identified with a different colour.

A multivariate principal coordinate analysis (PCoA) confirmed the grouping of genotypes based on UPGMA. The first two axes explain 51.35% of the genetic variation. The first component explains 33.75% of the variation (Figure 9) and clearly separate *T. patula* from *T. erecta* and *T. tenuifolia*, which are spread in the same area of the PCoA plot. The second component explains 17.61% of the total variation and allows a better separation of the accessions within the species. The groups observed agree with the ones detected in the UPGMA dendrogram. Among *T. patula* individuals, Szinkeverek is the most genetically different. The three individuals of *T. erecta* are very dispersed, indicating a wide genetic diversity. The individuals of *T. tenuifolia* can be separated into two subgroups (Figure 9). The first one is composed by Sarga and Luna Orange, and the second includes Luna Lemon, Luna Root and Luna Gold.

## 3. Discussion

### 3.1. Morphological Traits of the Seeds and Germination

The study of the main characteristics of seeds is important for identifying different cultivars and checking whether these characteristics of the seeds may influence other traits of interest. Various significant phenotypic correlations were identified between seed characters and other characters analysed in this study, including plant growth, flowering, and fruiting. In contrast, they did not influence seed germination and plant emergence (a single significant correlation for an alpha level of 0.05 was identified between seed width and emergence). In the three species, except for a few cultivars (i.e., cv. Arctic of *T. erecta* species, with very low germination), all cultivars showed adequate germination capacity. However, the optimal use of substrates can ensure both the proper germination of seeds as well as the good rooting and growth of plants and, subsequently, good decorative traits [46].

A high rate of seed germination is extremely important because marigolds are directly sown in the field, which is more practical and economical for producers compared to transplanting. Using sowing, labour costs are significantly reduced in the absence of supplementary costs by propagation or transplantation [29]. However, some shortcomings are manifested in direct sowing in the soil in the form of uneven germination and slow emergence, resulting in a poor stand establishment. These deficiencies are amplified if sowing is performed in improper conditions, i.e., hot or cold fields [29]. To overcome such problems, various treatments are proposed, including seed priming (seed hydration, followed by the redrying process). This treatment has proven to be a straightforward and cost-effective pre-sowing procedure that can ensure good results in marigolds [47,48]. In addition to seed priming, ascorbate and salicylic acid treatments applied to seeds were effective to ameliorate the adverse effects of salinity in *T. patula* [49].

### 3.2. Vegetative and Reproductive Traits and Phenophases

Analysis of vegetative and reproductive traits and phenophases often indicated significant differences between the three species. *T. erecta* cultivars were noted for the growth and vigour of the plants. They had a much higher plant height compared to cultivars of *T. tenuifolia* and *T. patula* but had the smallest number of flowers and the largest floral heads. *T. tenuifolia* had the smallest height, the largest number of branches and the largest number of flower heads but were much smaller compared to the cultivars of the other two species. Plant phenology is strongly influenced by genetic and environmental factors and also by the interaction of the genotype and environmental and crop factors [50,51]. Because the growth conditions were virtually identical for all cultivars included in this study, cultivars with traits of interest and adequate response under the ecologically given conditions could be identified. The purpose of the investigation was twofold. First, to identify and recommend cultivars with adequate decorative value, which would be suitable in similar ecological conditions of the area (N–W of Romania); second, to identify possible parental forms for new breeding works. In the study, amongst the earliest cultivars, as phenological stages, were Luna Orange, Luna Lemon, Luna Rot, and Luna Gold, all belonging to *T. tenuifolia*, and the later cultivar was Orion of *T. patula*. The data registered indicated that the shortest life cycle is found in *T. tenuifolia* and is more extensive in *T. erecta* and *T. patula*. In another study on *T. patula* cultivars, the late-flowering length cycle induced higher plants and thicker stems, more developed aerial parts and roots, and bigger inflorescences but a shorter flowering duration period. Cultivars with a precocious cycle length formed more inflorescences and had a longer flowering period [52].

The flowering period is of exceptional importance when choosing ornamental plants for green areas. Marigolds need about 45 days from sowing to flowering, and in African or American marigolds (*T. erecta*), the time required is longer than for French marigolds (*T. patula*) [3]. In our experience, 45 days after sowing, the cultivars of *T. tenuifolia* were already at the time of full flowering, but in *T. patula* and especially *T. erecta*, this phenophase was realised much later.

In any case, marigolds are robust plants, are non-fussy and can be easily grown [3]. It seems that the flowering duration is relatively short because, in most genotypes, the flowering lasts about 21–22 days as the mean of the analysed species. It is possible that this was also because no fertilisers were applied, and fertilisers, i.e., potash fertilisers, prolong the flowering period [53,54]. Additionally, the plant and flower characteristics were not influenced by cultural and physical practices or treatments because, to avoid any influences on some characteristics, in the experience, no interventions were performed on the plants. Moreover, it is known that except for fertilisers, different operations, such as pinching off the first flowers before they open, can increase the number of flowers per plant [3]. In African marigolds, topping combined with gibberellic acid (using a concentration of 100 mg L^−1^) increased flowering and other traits of interest [55].

The results confirm that African marigolds are suitable cultivars as bedding plants, and Lemon marigolds (*T. tenuifolia*) as ornamental bedding and pot plants; in contrast, French marigolds cultivars are proper for edging flowerbeds, mass plantings or to be cultivated in containers and window boxes [3,28].

### 3.3. Usefulness of the Statistical and Molecular Methods Used in the Evaluation and Selection of Genotypes

The use of box plot diagrams offers the possibility of obtaining the high graphical and visual impact of the data, which helps to define the upper and lower limit of traits, beyond which any data will be considered outliers or aberrant values. These outliers are also called extreme because they are at the end of a data series. They can be either very small or large and can, therefore, affect the general observations, the final results or even the conclusions of the study [56]. In addition, boxplots can also be used as the tool to select varieties in the DUS test of UPOV because they can adequately reflect the uniformity and stability of the TGP/1/7 characteristics [57]. In our data, outliers for several traits have been identified. In presenting the results, outliers inferior to seed germination were highlighted, both in *T. patula* (Mr. Majestic) and *T. erecta* (Arctic). Obviously, because they entered the calculation of the average values, these extreme values greatly influenced, in a negative sense, the final result for the two species. In the case of plant height, there was an upper outlier in *T. patula* (Queen Sophia) and a lower outlier in *T. erecta* (Antigua Orange).

Usually, in data analyses, once such outliers are identified, they can be removed from the data series before moving on to further analysis (statistical calculations). In this way, accurate results can be obtained, uninfluenced by any extreme or abnormal values. In plant breeding, because the diversity may be of interest for the possible identification and selection of parental forms to be used in artificial hybridisations [58,59,60], the outliers were not excluded in our study. However, it can be assumed that the cultivars with very poor germination were a mistake of the producing companies that introduced poor-quality seeds in the market or of staff who had to verify the fulfilment of the quality standards before accepting the entry on the market of lower-quality seeds, below the accepted standards for germination capacity. In the case of other traits, such as plant height, the two deviations recorded as aberrant values in *T. patula* and *T. erecta* may also reflect errors not allowed in the identification or labelling of the seeds. This is because the two cultivars appear to have aberrant height compared to the height of the plants in the species in which they were included. Such hypotheses can also be analysed based on the results provided by multivariate analysis, especially on phylogenetic relationships, and the classification of a cultivar in the species pattern using specific molecular techniques, particularly molecular markers.

Pearson correlations highlighted different positive or negative relations amongst the evaluated traits. Some correlations indicated that plants that produced larger flowers required more prolonged periods for flowering and fruit set, and plants with numerous, smaller heads would produce smaller seeds. There was a positive correlation between seed size and duration of reproductive phenological stages, implying that cultivars that require longer time for flowering and fruit formation will produce larger seeds. Such correlations can be useful in horticultural practice and marigolds breeding [60]. Based on close correlations between different characters, it is possible to establish how to increase the efficiency of marigolds crops by applying technical and cultural operations (for example, fertilisation, interventions on plants to increase the number of flowers per plant or flower size) [61]. If some phenotypic correlations are also accompanied by significant genetic correlations, they could be efficiently used in marigolds breeding as indices of indirect selection. Consequently, selection based on important attributes and their correlations would result in a useful genetic advance in marigold breeding, including interspecific hybridisations between *T. erecta* and *T. patula* [62,63,64,65].

Molecular analyses using different techniques have been successfully used in various horticultural species, including *Tagetes*, to estimate genetic diversity [39,40,66] or to detect homonymous and synonymous genotypes [67]. Molecular markers have become a valuable tool for the identification of hybrid purity, genetic diversity and characterisation of germplasm. In addition, if markers are related to the traits of interest, they provide a means of effective selection of the plants having the desirable traits [39,40]. Information on molecular variation in *Tagetes* species is very useful for further molecular breeding studies, constructing genetic maps, and genetic conservation [68]. Analysis of phylogenetic relationships and fingerprinting of different genotypes can be used to select materials in the species of *Tagetes*. Additionally, these studies are relevant in breeding marigolds to obtain hybrids that exhibit an intense heterosis effect for desirable characters and can assure an extremely efficient selection in obtaining new cultivars.

## 4. Materials and Methods

### 4.1. Plant Material

**Figure 10:**
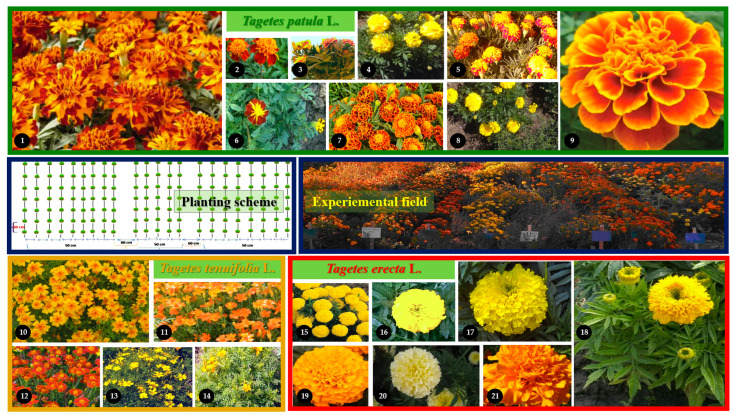
Planting scheme, experimental field and analysed cultivars belonging to three species of *Tagetes*: *T. patula* (1—Bolero, 2—Orange Flame, 3—Szinkeverek, 4—Robuszta, 5—Orion, 6—Mr. Majestic, 7—Cresto Flame, 8—Cresto Yellow, 9—Queen Sophia), *T. tenuifolia* (10—Luna Gold, 1—Luna Orange, 12—Luna Rot, 13—Luna Lemon, 14—Sarga) and *T. erecta* (15—Aranysarga, 16—Alacsony Citromsarga, 17—Magas Citromsarga, 18—Cupid Golden Yellow, 19—Antigua Orange, 20—Artic, 21—Hawaii).

Nine cultivars of *T. patula* (Bolero, Orange Flame, Szinkeverek, Robuszta, Orion, Mr. Majestic, Cresto Flame, Cresto Yellow, and Queen Sophia), five of *T. tenuifolia* (Luna Gold, Luna Orange, Luna Rot, Luna Lemon, and Sarga) and seven of *T. erecta* (Aranysarga, Alacsony Citromsarga, Magas Citromsarga, Cupid Golden Yellow, Antigua Orange, Artic, and Hawaii) were used for the present study (Figure 10). 

### 4.2. Analysis of Seed Characteristics and Seed Germination

The following seed characteristics were analysed for all cultivars: seed length, width and thickness. Seed germination was performed using International Seed Testing Association (ISTA) rules [69], using four replicates of 100 seeds per sample. Seed germination was conducted in Petri dishes on double filter paper discs and cotton moistened with distilled water. Germination was performed in a germination chamber at 23 °C, with a 16 h photoperiod.

### 4.3. Description of the Study Site, Phenological Studies and Morphologic Traits Analysed

A micro-culture (Figure 10) was established in Cluj-Napoca, N-W Romania (with an average annual temperature of 8.2 °C and an average annual rainfall of 560 mm). Total porosity was comprised between 35–90 cm and aeration porosity between 35–70 cm; the wilting coefficient was between 0–15 cm; field capacity was moderate throughout the profile, useful water capacity was between 0–20 cm, and mean suitability values between 0–20 cm. The favorability of the soil and the general conditions in the field experiment were specific to a characteristic area of the Someș Mic Valley Corridor [70].

The establishment of the micro-culture was achieved by direct sowing on the land prepared by ploughing and disking, followed by levelling the ground in the spring. The sowing took place in April, with a mean temperature of 12 °C; the maximum temperature registered was 36 °C (August), and the minimum of 10 °C was recorded in October. Planting was done manually; rows were traced using the marker, respecting distances of 60 cm between the different species and cultivars sown, 50 cm between rows and 30 cm between plants in the row. The seeds were sown at a depth of 2–3 cm.

Throughout the experiment, several main phenological stages were followed: full emergence, full flowering, full maturation of achenes (‘seeds’), duration of full flowering [71]. The following morphological traits were analysed: plant height, stem diameter at the height of 5 cm above ground level, number of the main branches per stem, angle of branch insertion, number of floral heads, the diameter of floral heads, and diameter of the disc. Branch insertion angle was estimated as the mean, based on the angle of all the branches and stem of the plant, respectively the angle between the vertical axis and the normal angle of the fitted circle.

Observations on plants and flowers were recorded at the time of full flowering. For all plant traits, 30 plants per cultivar were analysed (considering three replicates, with ten plants per replication), the data being subsequently processed as average values. The coefficient of variation (CV%) was calculated using individual plant data.

Based on the results obtained in the evaluation of phenotypic traits, several genotypes of the three species were selected for the genetic analysis. Priority was given to the cultivars with favourable ornamental characteristics, considered appropriate or potential parents to be used in artificial crosses in new breeding works.

### 4.4. Genetic Diversity Analysis

Genomic DNA was extracted from 13 cultivars from the three species. For each sample, DNA was obtained from ca. 100 mg of young leaf tissue using the CTAB method with some modifications [72]. After electrophoresis on a 1.0% agarose gel, DNA concentration was quantified using a Nanodrop ND-1000 (Nanodrop Technologies, Wilmington, DE, USA) spectrophotometer. Samples were adjusted to a DNA concentration of 20 ng/L. The quality of DNA was evaluated through the 260/280 nm and 260/230 nm absorbance ratios [73]. Six genomic highly polymorphic SSR markers developed by Whankaew et al. [40] were used to screen the genotypes under study. An M13-tailed forward primer was used in combination with a standard M13 primer dye-labelled with FAM, NED, or VIC fluorophores at its 5′-end. PCR amplifications were performed in a total volume of 12 μL with 20 ng of DNA, 1.5 mM MgCl_2_, 0.05 M of forward primer, 0.25 M of reverse primer, 0.2 M of fluorescent M-13 primer, 0.2 mM dNTPs, and 0.20 μL Taq DNA polymerase (TaKaRa Bio Inc., Kyoto, Japan) at a concentration of 5 U/μL. Amplifications were carried out in an Eppendorf Mastercycler ep gradient S (Eppendorf AG, Hamburg, Germany) thermocycler. Amplification procedure via the thermocycler consisted of an initial step at 94 °C for 5 min; 35 cycles of 94 °C for 30 s, 30 s at 62 °C, and 72 °C for 45 s; and final 10 min extension at 72 °C. PCR products were diluted in formamide and analysed on an automated DNA sequencer ABI PRISM 3100-Avant with a GeneScan 600LIZ (Applied Biosystems, Foster City, CA, USA) size standard. The data were analysed using the GeneScan software (Applied Biosystems) to obtain the electropherograms and polymorphisms were analysed with Genotyper DNA Fragment Analysis software (Applied Biosystems). 

For the genetic characterisation of the 13 *Tagetes* cultivars, the number of polymorphic alleles (Na), the number of genotypes (NG), the observed heterozygosity (Ho), the expected heterozygosity (He) and the polymorphic information content (PIC) were determined for each SSR locus using PowerMaker software [74]. A UPGMA dendrogram with 1000 bootstrap randomisation was performed using the function aboot from the R package poppr v2.8.2. Principal coordinates analysis (PCoA) was performed to represent graphically the genetic relationships among individuals using GenAlEx 6.5 software. 

### 4.5. Data Analysis

The mean values of the traits were analyzed using the ANOVA test, and if the null hypothesis was rejected, a significance test was performed. To analyse the differences between the means, the Duncan test was used as a posthoc test (*p* < 0.05). Data were analysed using Statgraphics Centurion v.16 (Statgraphics Technologies, Inc., The Plains, VA, USA), and boxplots were realised using Microsoft Excel (2018).

The phenotypic correlation among the pairs of the studied traits was analysed by calculating Pearson coefficients of correlation using cultivar means. In addition, a principal components analysis (PCA) based on standardized Euclidean distances was performed using data for the traits evaluated in order to globally evaluate the variation of the sets of cultivars from the three different species. The PCA multivariate technique was chosen over canonical variate analysis (CVA) as our objective is to evaluate the relationships among varieties independently of the species they belong to. In contrast, CVA minimizes the within-species variation and maximises between-species variation, which is not the objective of our study; this is more appropriate for taxonomical purposes. The dendrogram of cluster analysis of the analysed traits of the *Tagetes* cultivars was performed using the single linkage method, the Gover similarity index. Pearson coefficients of correlation, PCA and dendrogram were performed using Past software [75].

## Figures and Tables

**Table 1:** Main seed traits and their coefficient of variation for the *Tagetes* cultivars and the germination capacity of the seeds.

No	Genotype	Seed Length		Seed Width		Seed Thickness		Germination
		(mm)	CV%	(mm)	CV%	(mm)	CV%	(%)
*T. patula*								
1	Bolero	8.8 e	22.3	2.8 ab	16.3	2.4 a	12.6	78.3 d
2	Orange Flame	11.3 cd	2.8	2.7 ab	21.4	2.3 ab	20.1	100.0 a
3	Szinkeverek	11.5 bcd	4.1	2.4 bc	10.2	2.3 ab	9.7	97.4 ab
4	Robuszta	13.7 abc	2.5	2.5 abc	9.6	1.7 ab	8.0	100.0 a
5	Orion	14.0 a	17.5	3.2 a	13.6	2.4 a	8.6	100.0 a
6	Mr. Majestic	13.0 abcd	2.7	2.3 bc	7.8	0.8 c	14.9	43.5 e
7	Cresto Flame	12.0 abcd	1.6	2.5 abc	7.6	2.4 a	4.7	87.0 c
8	Cresto Yellow	11.0 de	2.2	1.9 c	9.9	1.5 bc	7.7	91.3 bc
9	Queen Sophia	13.9 ab	1.1	2.3 bc	4.7	2.1 ab	7.8	92.2 bc
*T. tenuifolia*								
10	Luna Gold	7.7 ab	1.2	1.6 a	1.4	1.4 ab	4.2	100.0 a
11	Luna Orange	8.8 a	0.8	0.9 b	1.1	1.1 b	6.3	88.7 bc
12	Luna Rot	6.8 b	1.0	1.6 a	1.2	1.2 ab	5.9	97.6 ab
13	Luna Lemon	6.6 b	1.7	1.4 ab	1.1	1.1 b	11.0	100.0 a
14	Sarga	7.2 ab	2.2	1.6 a	1.6	1.6 a	2.8	87.0 c
*T. erecta*								
15	Aranysarga	10.9 c	1.3	2.6 ab	2.5	1.5 d	5.8	93.0 a
16	Alacsony Citromsarga	14.5 a	0.9	3.0 a	4.5	2.4 ab	4.5	65.2 b
17	Magas Citromsarga	12.3 bc	1.1	2.2 b	4.8	2.1 c	7.4	100.0 a
18	Cupid Golden Yellow	14.9 a	1.2	2.9 a	5.3	2.5 a	4.0	97.4 a
19	Antigua Orange	15.1 a	1.1	2.6 ab	4.3	2.2 bc	5.5	95.7 a
20	Artic	13.0 ab	0.9	2.6 ab	5.2	2.3 abc	7.3	8.7 c
21	Hawaii	13.2 ab	1.6	2.3 b	6.9	2.3 abc	4.9	95.7 a

Means in columns for the cultivars of each species followed by the same letter are not significantly different at the *p* < 0.05 level according to Duncan’s multiple range test (DMRT).

**Table 2:** Main plant traits and their coefficient of variation of the *Tagetes* cultivars.

No	Genotype	Plant Height		Stem Diameter		No. Branches/Stem		Angle of Branch Insertion	
		(cm)	CV%	(mm)	CV%	(n)	CV%	Degree	CV%
*T. patula*									
1	Bolero	25.4 b	11.3	10.5 bc	0.8	8.7 b	1.7	49.5 c	2.7
2	Orange Flame	23.3 b	10.5	8.5 d	1.0	10.7 ab	10.5	60.2 ab	1.8
3	Szinkeverek	26.2 b	6.3	11.5 ab	4.0	8.9 b	15.4	40.0 d	4.7
4	Robuszta	22.1 b	13.1	12.9 a	3.2	13.7 a	13.3	59.4 ab	1.3
5	Orion	26.0 b	6.4	9.4 cd	2.7	7.6 b	27.4	59.9 ab	1.4
6	Mr. Majestic	20.7 b	11.6	9.3 cd	4.7	7.6 b	11.7	60.6 a	2.3
7	Cresto Flame	25.3 b	16.2	11.3 b	3.3	9.2 b	9.0	50.0 c	1.6
8	Cresto Yellow	25.4 b	5.6	11.6 ab	4.4	9.6 b	11.8	51.3 c	0.9
9	Queen Sophia	47.8 a	3.1	11.2 b	5.7	10.7 ab	18.8	53.4 bc	2.7
*T. tenuifolia*									
10	Luna Gold	21.8 a	5.0	8.7 b	8.2	16.0 b	8.8	52.4 ab	3.9
11	Luna Orange	21.0 b	6.3	9.1 ab	10.1	16.4 ab	7.5	52.2 ab	3.9
12	Luna Rot	21.1 b	3.7	9.2 a	7.7	16.9 a	5.5	52.8 a	3.0
13	Luna Lemon	21.3 ab	4.7	9.2 a	6.1	16.7 ab	14.1	51.5 b	2.7
14	Sarga	21.0 b	5.8	9.2 a	7.3	16.0 b	8.8	51.6 b	2.0
*T. erecta*									
15	Aranysarga	67.2 ab	6.8	14.8 ab	9.5	13.6 a	9.8	66.5 ab	3.1
16	Alacsony Citromsarga	69.8 a	4.8	15.8 a	6.6	14.2 a	11.0	71.1 a	1.3
17	Magas Citromsarga	59.7 b	3.0	15.4 ab	4.0	10.4 bc	11.8	69.8 a	1.2
18	Cupid Golden Yellow	59.9 ab	2.4	12.6 abc	3.4	8.9 c	10.4	59.8 bc	1.4
19	Antigua Orange	24.4 c	4.6	8.9 c	3.9	11.8 ab	13.3	37.3 c	1.8
20	Artic	60.6 ab	2.2	11.6 bc	4.2	10.0 bc	14.4	65.1 ab	1.9
21	Hawaii	65.8 ab	3.7	13.0 ab	2.1	12.1 ab	22.0	60.5 bc	1.8

Means in columns for the cultivars of each species followed by the same letter are not significantly different at the *p* < 0.05 level according to Duncan’s multiple range test (DMRT).

**Table 3:** Main traits of the flowers, number of fruits per plant and their coefficient of variation for the *Tagetes* cultivars.

No	Genotype	No. Flowers		Corolla Diameter		Disc Diameter		No. Fruits	
		(no)	CV%	(cm)	CV%	(cm)	CV%	(no)	CV%
*T. patula*									
1	Bolero	28.3 bc	7.1	3.4 d	15.8	1.2 b	12.6	5.2 cd	20.9
2	Orange Flame	29.7 bc	5.3	5.0 ab	5.5	1.5 ab	6.6	13.1 a	9.7
3	Szinkeverek	25.4 c	10.0	3.8 cd	7.3	1.2 b	20.1	13.0 a	14.9
4	Robuszta	30.0 abc	7.6	3.0 d	6.6	1.3 ab	22.7	4.8 d	29.2
5	Orion	29.7 bc	5.6	5.4 a	4.6	1.7 a	9.1	12.2 ab	9.8
6	Mr. Majestic	35.7 ab	3.7	3.1 d	5.1	1.1 b	14.4	10.4 ab	18.0
7	Cresto Flame	25.9 c	4.5	4.5 abc	3.6	1.3 ab	13.3	8.7 bcd	18.2
8	Cresto Yellow	25.9 c	3.0	4.4 bc	4.4	1.3 ab	9.9	9.2 abc	25.3
9	Queen Sophia	38.9 a	4.0	3.2 d	6.6	1.4 ab	8.9	9.6 abc	18.2
*T. tenuifolia*									
10	Luna Gold	91.9 b	2.8	0.9 b	20.1	0.5 a	18.9	35.1 ab	13.3
11	Luna Orange	92.1 ab	1.1	1.0 a	13.5	0.5 a	17.3	36.0 a	9.0
12	Luna Rot	92.2 ab	2.2	1.0 a	15.0	0.5 a	17.4	32.6 ab	6.2
13	Luna Lemon	92.2 ab	1.5	1.0 a	16.0	0.5 a	18.9	32.3 b	7.4
14	Sarga	96.4 a	3.8	1.0 a	16.4	0.5 a	16.2	32.1 b	7.7
*T. erecta*									
15	Aranysarga	26.6 ab	12.8	8.5 ab	2.5	4.5 b	3.3	8.4 abc	28.5
16	Alacsony Citromsarga	32.7 a	4.8	9.0 ab	4.4	4.2 b	6.6	10.9 a	17.5
17	Magas Citromsarga	16.7 abc	6.0	7.9 bc	4.3	5.0 ab	5.1	8.8 abc	11.1
18	Cupid Golden Yellow	26.6 ab	4.7	6.4 c	1.9	4.1 b	3.4	9.1 ab	13.9
19	Antigua Orange	5.3 c	16.2	10.1 a	2.0	7.7 a	2.7	2.6 d	28.4
20	Artic	8.6 bc	15.6	7.1 bc	2.6	4.2 b	2.5	4.4 cd	32.0
21	Hawaii	14.4 abc	9.9	7.5 bc	1.9	4.5 b	2.5	6.0bcd	36.3

Means in columns for the cultivars of each species followed by the same letter are not significantly different at the *p* < 0.05 level according to Duncan’s multiple range test (DMRT).

**Table 4:** Time (d) since sowing to reach the main phenological phases.

No	Genotype	Phenological Phases (in Days)							
		Seedling Emergence	Stem Formation	Branching	Budding	Full Flowering	Flowering Duration	Fruit Formation	Fruit Ripening
*T. patula*									
1	Bolero	14 ab	24 b	33 b	45 b	63 b	24 ab	78 b	98 bc
2	Orange Flame	16 ab	29 ab	36 ab	46 b	65 b	17 cd	81 ab	106 abc
3	Szinkeverek	16 ab	30 ab	40 ab	55 ab	70 ab	25 a	88 ab	106 abc
4	Robuszta	16 ab	30 ab	40 ab	53 ab	70 ab	22 ab	84 ab	104 bc
5	Orion	27 a	45 a	60 a	77 a	100 a	26 a	11 8a	145 a
6	Mr. Majestic	10 b	19 b	25 b	33 b	41 b	15 d	54 b	67 c
7	Cresto Flame	15 ab	25 b	38 ab	51 ab	69 ab	20 bc	85 ab	107 ab
8	Cresto Yellow	14 ab	24 b	37 ab	50 ab	60 b	22 ab	75 b	95 bc
9	Queen Sophia	14 ab	23 b	29 b	40 b	56 b	20 bc	72 b	98 bc
*T. tenuifolia*									
10	Luna Gold	14 ab	24 ab	29 ab	36 ab	46 ab	21 a	62 ab	84 ab
11	Luna Orange	14 ab	22 b	28 b	37 a	45 b	21 a	61 b	83 b
12	Luna Rot	13 b	25 a	30 a	35 b	47 a	21 a	63 a	85 a
13	Luna Lemon	15 a	25 a	29 ab	36 ab	46 ab	21 a	62 ab	84 ab
14	Sarga	14 ab	24 ab	29 ab	36 ab	46 ab	20 b	62 ab	84 ab
*T. erecta*									
15	Aranysarga	24 a	39 a	51 a	65 a	77 ab	22 ab	92 abc	116 b
16	Alacsony Citromsarga	16 ab	31 b	42 ab	57 bc	71 b	22 ab	88 c	114 b
17	Magas Citromsarga	14 b	29 b	40 b	56 c	70 b	21 bc	88 c	114 b
18	Cupid Golden Yellow	24 a	34 ab	44 ab	61 ab	83 a	24 ab	98 ab	122 a
19	Antigua Orange	16 ab	31 b	40 b	61 ab	78 ab	25 a	94 abc	119 ab
20	Artic	14 b	29 b	40 b	57 bc	73 b	18 c	89 bc	115 b
21	Hawaii	19 ab	34 ab	46 ab	64 a	83 a	25 a	99 a	124 a

Means in columns for the cultivars of each species followed by the same letter are not significantly different at the *p* < 0.05 level according to Duncan’s multiple range test (DMRT).

**Table 5:** Phenotypic correlations between the pairs of traits analysed (below the diagonal) and the *p*-value of the correlation (above the diagonal). Correlations were calculated from the mean values of each of the 21 cultivars characterised.

Correlated Traits	1	2	3	4	5	6	7	8	9	10	11	12	13	14	15	16	17	18	19	20
1 Seed length		0.000	0.005	0.412	0.026	0.039	0.003	0.231	0.000	0.000	0.002	0.000	0.102	0.034	0.009	0.001	0.000	0.406	0.001	0.001
2 Seed width	0.746		0.000	0.452	0.059	0.073	0.001	0.183	0.000	0.001	0.027	0.000	0.016	0.003	0.001	0.000	0.000	0.255	0.000	0.000
3 Seed thickness	0.586	0.757		0.992	0.050	0.116	0.012	0.754	0.001	0.004	0.033	0.002	0.056	0.018	0.005	0.001	0.000	0.044	0.000	0.000
4 Germination	−0.189	−0.174	0.002		0.278	0.762	0.276	0.167	0.306	0.470	0.583	0.275	0.139	0.332	0.550	0.768	0.676	0.020	0.624	0.590
5 Plant height	0.486	0.418	0.434	−0.248		0.000	0.598	0.001	0.028	0.000	0.001	0.040	0.089	0.041	0.027	0.013	0.023	0.547	0.023	0.012
6 Stem diameter	0.452	0.400	0.354	−0.070	0.817		0.564	0.003	0.020	0.003	0.021	0.017	0.241	0.098	0.029	0.018	0.042	0.362	0.044	0.044
7 No. branches/stem	−0.619	−0.690	−0.535	0.249	−0.122	−0.133		0.887	0.000	0.082	0.380	0.000	0.369	0.331	0.093	0.039	0.018	0.768	0.023	0.060
8 Branches insertion	0.273	0.302	0.073	−0.313	0.673	0.617	−0.033		0.377	0.108	0.316	0.368	0.300	0.160	0.142	0.233	0.306	0.189	0.325	0.250
9 No. flowers	−0.822	−0.797	−0.670	0.235	−0.478	−0.504	0.769	−0.203		0.000	0.001	0.000	0.200	0.042	0.004	0.000	0.000	0.351	0.000	0.001
10 Corolla diameter	0.735	0.685	0.597	−0.167	0.708	0.614	−0.389	0.361	−0.810		0.000	0.000	0.052	0.004	0.001	0.000	0.000	0.195	0.000	0.000
11 Disc diameter	0.636	0.483	0.466	−0.127	0.657	0.499	−0.202	0.230	−0.664	0.928		0.004	0.142	0.022	0.015	0.001	0.003	0.162	0.003	0.002
12 No. fruits	−0.810	−0.813	−0.627	0.250	−0.450	−0.513	0.746	−0.207	0.972	−0.745	−0.602		0.272	0.086	0.013	0.001	0.001	0.429	0.001	0.004
13 Emergence	0.367	0.520	0.423	0.334	0.380	0.267	−0.207	0.238	−0.291	0.429	0.332	−0.251		0.000	0.000	0.000	0.000	0.004	0.000	0.000
14 Stem formation	0.465	0.623	0.509	0.222	0.450	0.370	−0.223	0.318	−0.448	0.606	0.497	−0.383	0.919		0.000	0.000	0.000	0.004	0.000	0.000
15 Branching	0.556	0.687	0.585	0.138	0.483	0.476	−0.376	0.332	−0.599	0.680	0.521	−0.533	0.866	0.961		0.000	0.000	0.004	0.000	0.000
16 Budding	0.675	0.724	0.670	0.068	0.535	0.511	−0.454	0.272	−0.723	0.787	0.659	−0.652	0.793	0.909	0.972		0.000	0.003	0.000	0.000
17 Full flowering	0.697	0.786	0.767	0.097	0.495	0.447	−0.510	0.235	−0.739	0.747	0.617	−0.680	0.793	0.893	0.948	0.979		0.003	0.000	0.000
18 Flowering duration	0.191	0.260	0.443	0.502	0.139	0.209	−0.069	−0.299	−0.214	0.295	0.317	−0.182	0.601	0.599	0.597	0.619	0.616		0.002	0.003
19 Fruit formation	0.678	0.769	0.779	0.114	0.493	0.444	−0.494	0.226	−0.718	0.741	0.611	−0.650	0.788	0.896	0.948	0.978	0.997	0.630		0.000
20 Fruit ripening	0.652	0.732	0.790	0.125	0.537	0.444	−0.417	0.262	−0.672	0.748	0.633	−0.600	0.784	0.894	0.931	0.960	0.982	0.609	0.989	

Correlation is significant at the level of *p* < 0.05; 0.01; 0.001 (2-tailed).

**Table 6:** SSR markers, forward (F) and reverse (R) probes, repeat motif, number of genotypes (NG), number of alleles per locus (Na), expected heterozygosity (He), observed heterozygosity (Ho) and polymorphism information content (PIC) in the set of 13 *Tagetes* cultivars.

Name	Sequences (5′–3′)		Repeat Motif	NG	Na	He	Ho	PIC
TE11	F	CGCCTAATTTGTTGCATGG	(AG)19	3	2	0.20	0.08	0.18
	R	ATGTCACCGCCAAAGGATT						
TE35	F	ACCCTCCTTGACCCTGTTG	(CT)15	2	2	0.47	0.00	0.36
	R	GGTGTTGTTGCTGCTTGCT						
TE38	F	CGGAAACGAACGAGTGAAGT	(TA)7	4	3	0.38	0.09	0.34
	R	GCGTATAAAATCCCTGCCCT						
TE59	F	CCGGTTTGTGAAATCTGAAG	(AG)9	6	6	0.66	0.23	0.60
	R	CACAGCTAAACTCACGCACA						
TE70	F	AATCAGCCATTATCAACCCT	(AG)24	3	3	0.52	0.00	0.44
	R	CAACTCATGTTTCACCCAAA						
TE78	F	GAGAGGATCTGGTGGGATGA	(CT)7	4	5	0.53	0.20	0.41
	R	GGTGGCTCCAAACTACCAAG						

## Data Availability

Not applicable.

## Supplementary Materials

The following supporting information can be downloaded at: https://www.mdpi.com/article/10.3390/plants11060760/s1, Table S1: Pairwise Nei genetic distances among 13 cultivars of *Tagetes* from three different species (*T. patula*, *T. tenuifolia*, and *T. erecta*) using six SSR markers.
