# Supplementary material for: Biological Traits and Genetic Relationships Amongst Cultivars of Three Species of Tagetes (Asteraceae)
Source: Plants (Basel). 2022 Mar 12;11(6):760. doi: 10.3390/plants11060760 (PMC8951573; doi:10.3390/plants11060760)
Supplement: Supplementary file 1 [file plants-11-00760-s001.zip › plants-1604669-supplementary.pdf]

Table S1. Pairwise Nei genetic distances among 13 cultivars of *Tagetes* from three different species (*T. patula*, *T. tenuifolia*, and *T. erecta*) using six SSR markers (Table 6).

	T. patula					*T. tenuifolia*					*T. erecta*		
	Bolero	Orange Flame	Szinkeverek	Robuzsta	Orion	Luna Gold	Luna Orange	Luna Rot	Luna Lemon	Sarga	Aranysarga	Magas Citromsarga	Cupid Golden Yellow
T. patula													
Bolero	0.0												
Orange Flame	7.0	0.0											
Szinkeverek	11.0	8.0	0.0										
Robuzsta	7.0	0.0	8.0	0.0									
Orion	5.0	4.0	12.0	4.0	0.0								
T. tenuifolia													
Luna Gold	10.3	12.3	12.3	12.3	10.3	0.0							
Luna Orange	13.3	14.3	14.3	14.6	14.3	6.8	0.0						
Luna Rot	11.3	14.3	14.3	14.3	10.3	4.0	7.8	0.0					
Luna Lemon	10.0	13.0	9.0	14.3	9.0	4.0	7.8	4.0	0.0				
Sarga	18.0	15.0	15.0	13.3	19.0	9.0	7.8	7.0	12.0	0.0			
T. erecta													
Aranysarga	13.0	10.0	10.0	12.7	14.0	8.2	7.5	9.1	7.5	5.5	0.0		
Magas Citromsarga	13.0	11.0	19.0	9.7	9.0	7.7	12.5	8.7	10.0	14.0	15.0	0.0	
Cupid Golden Yellow	19.0	16.0	16.0	14.7	20.0	9.7	9.5	9.7	13.0	7.0	8.0	11.0	0.0
